# [^18^F]FDG-PET/CT in DLBCL-patients treated with CAR-T cell therapy: potential for defining patient prognosis

**DOI:** 10.1016/j.ejro.2025.100663

**Published:** 2025-06-05

**Authors:** Helena A. Peters, Ben-Niklas Bärmann, Emil Novruzov, Daniel Weiss, Matthias Boschheidgen, Vivien Lorena Ivan, Nora Liebers, Johannes Fischer, Eduards Mamlins, Aleksandar Radujkovic, Guido Kobbe, Julian Kirchner, Peter Minko, Kathrin Nachtkamp, Paul Jäger, Christina Antke, Frederik L. Giesel, Sascha Dietrich, Gerald Antoch, Kai Jannusch

**Affiliations:** a Department of Diagnostic and Interventional Radiology, Medical Faculty and University Hospital Düsseldorf, Heinrich-Heine-University Düsseldorf, Düsseldorf D-40225, Germany; b Department of Nuclear Medicine, Medical Faculty and University Hospital Düsseldorf, Heinrich-Heine-University Düsseldorf, Düsseldorf D-40225, Germany; c Department of Hematology, Oncology and Clinical Immunology, Medical Faculty and University Hospital Düsseldorf, Heinrich-Heine-University Düsseldorf, Düsseldorf D-40225, Germany; d Center for Integrated Oncology Aachen Bonn Cologne Düsseldorf (CIO ABCD), Germany

**Keywords:** Diffuse large B cell lymphoma, CAR-T, FDG-PET/CT, Overall survival, Progression-free survival

## Abstract

**Objectives:**

The aim of this study is to evaluate the potential of [^18^F]FDG-PET/CT in terms of prognostic value and treatment monitoring in relapsed / refractory diffuse large B-cell lymphoma (DLBCL)-patients treated with chimeric antigen receptor T-cell (CAR-T) therapy.

**Material & methods:**

Forty-eight [^18^F]FDG-PET/CT scans, acquired at pre-defined time points (t_0_ – t_2_) of 18 DLBCL-patients (mean age: 60 ± 12 years) treated with CAR-T cell therapy were retrospectively enrolled. Median time of follow-up was ten months (IQR 6–16) following CAR-T cell infusion. SUV_max_, sum of the product of diameters (SPD), Deauville score (DS) and Lugano classification (LC) were evaluated. Clinical parameters (age, sex) were obtained. Survival time analyses for progression-free survival (PFS) and overall survival (OS) were calculated, the latter by using the Kaplan-Meier method and Cox regression including a hazard ratio (HR). *P* values below 0.05 were defined as statistically significant. 95 %-confidence intervals (CI) were calculated.

**Results:**

Patients with a SUV_max_> 9.0 at t_0_ (median as threshold value) had a significantly shorter PFS (*p* = 0.04) and OS (*p* < 0.01). According to LC, a progressive disease (PD) at t_1_ (*p* = 0.02) and t_2_ (*p* < 0.01) was correlated with a reduced OS. SUV_max_ > 9.0 at t_0_ (*p* = 0.03, HR = 7.0, CI: 1.3–40.5) and DS > 3 at t_1_ (*p* = 0.04, HR = 8.2, CI: 1.1–61.3) were associated with an increased risk of a PD.

**Conclusion:**

SUV_max_ of [^18^F]FDG-PET/CT seems to be useful as a prognostic marker in DLBCL-patients undergoing CAR-T cell therapy. Furthermore, scores of clinical established Deauville classification and Lugano response criteria acquired at post-CAR-T [^18^F]FDG-PET/CT might be an indicator for early therapy failure.

## Highlights

• Pretherapeutic SUV_max_ > 9 may be an indicator for shorter PFS and OS in DLBCL-patients undergoing CAR-T cell therapy.
• Deauville score and Lugano classification derived from post-therapeutic [^18^F]FDG-PET/CT could indicate early treatment failure.
• Identifying non-responders ideally before CAR-T cell therapy initiation could be crucial.

## Graphical Abstract

**Figure:**
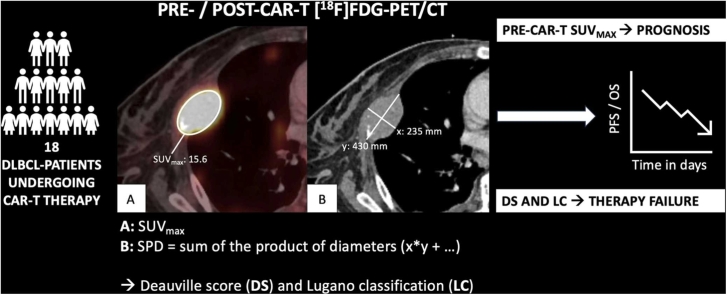

## 1 Introduction

Diffuse large B-cell lymphoma (DLBCL)-patients in a relapsed / refractory stage face low survival rates and poor prognosis, making the assessment of new therapeutic options crucial. The introduction of anti-CD19 chimeric antigen receptor T (CAR-T) cells in 2017 marked a significant advancement in patient-centered therapy [1], [2], [3], [4]. Current DLBCL studies examining CAR-T cell therapy reported higher durable response rates with complete response (CR) in up to 50–65 % of patients [5], [6], [7], [8].

Typically inserted by a lentiviral vector, a tumor antigen recognizing receptor - (CAR) and costimulatory domain genes are inserted in patients’ own (autologous) T-cells. During the treatment procedure, genetically modified autologous T-cells are reinfused after lymphodepleting chemotherapy [9]. Currently, there are six commercial CAR-T products available: axicabtagene ciloleucel, ciltacabtagene autoleucel, idecabtagene vicleucel, tisagenlecleucel, brexucabtagene autoleucel, and lisocabtagene maraleucel. Among these, tisagenlecleucel, axicabtagene ciloleucel, and lisocabtagene maraleucel are approved for DLBCL-patients in a relapsed / refractory stage [9]. Different immunological pathways are activated, which can affect the therapeutic response. In this setting imaging plays a pivotal role for definition of patient prognosis and therapy response assessment or resistance.

Over the past decade metabolic imaging, particularly ^18^F-Fluordeoxyglucose positron emission tomography-computed tomography ([^18^F]FDG-PET/CT), has become the reference imaging method for baseline, staging and monitoring of DLBCL-patients [10]. Before CAR-T cell therapy, it is important to perform baseline [^18^F]FDG-PET/CT imaging, usually at two time points, pre-leukapheresis and pre-lymphodepletion chemotherapy [11]. However, at our department only one baseline examination was conducted prior to lymphodepletion. Although there is no standardized guideline for imaging-based follow-up after CAR-T cell therapy, current literature emphasizes [^18^F]FDG-PET/CT examinations and subsequent evaluations at one month and 90–100 days after CAR-T cell infusion as optimal intervals for staging and monitoring therapeutic response, non-response, or therapeutic failure [12], [13]. Consequently, radiologists and nuclear medicine specialists play a pivotal role in the therapeutic monitoring of DLBCL-patients after CAR-T cell therapy.

A high total metabolic tumor volume (TMTV) is known to correlate with a poorer outcome in DLBCL-patients treated with guideline-centered chemotherapy [14]. Nonetheless, the prognostic potential of [^18^F]FDG-PET/CT in DLBCL-patients undergoing CAR-T cell therapy has not been sufficiently explored [15], [16]. Initial studies have already indicated a correlation between TMTV and poorer patient outcomes [12], [17], [18], [19]. However, in daily routine determination of TMTV is very time-consuming and no standard procedure for staging and definition of patient prognosis. Easily accessible SUV_max_ values might be a promising alternative for clinical routine. There are already individual studies [19], [20], [21], [22] like Cohen et al. (2022) who were able to demonstrate that the pre-therapeutic SUV_max_ may be a valid parameter for risk stratification of patients treated with CAR-T cell therapy [22]. Nonetheless, SUV_max_ of [^18^F]FDG-PET/CT has not yet been sufficiently investigated, especially with regard to its prognostic potential for therapeutic response and survival. Furthermore, Deauville score (DS) and the Lugano classification (LC) as widely used and clinical experienced report scales might be helpful in DLBCL-patients to identify early therapy failure after CAR-T cell therapy.

Thus, this study aims to elucidate the prognostic potential of [^18^F]FDG-PET/CT imaging markers which are easily accessible in clinical routine. Furthermore, standard reporting scales (DS / LC) assessed at pre-defined time points in DLBCL-patients receiving CAR-T cell therapy are evaluated concerning their potential in therapy response assessment and definition of patient prognosis.

## 2 Material and methods

### 2.1 Patients

This retrospective study was approved by the institutional review board of the University Düsseldorf (study number: 2023–2618) and it was performed in accordance with the Declaration of Helsinki [23].

This study retrospectively enrolled the data of 48 [^18^F]FDG-PET/CT scans (18 patients) acquired at three different time points (t_0_ [18 (IQR 9–48) days before CAR-T cell infusion and 14 (IQR 10–41) days before lymphodepletion], t_1_ [30 days (IQR 28–36) after CAR-T cell infusion], and t_2_ [135 days (IQR 92–179) afterwards]) between 06/2020 and 11/2023. In accordance with the institutional review board, written informed consent was waived because of the retrospective study design. Inclusion criteria were defined as follows: (i) age above 18 years, (ii) CD19-targeted CAR-T cell therapy for relapsed or refractory DLBCL, (iii) no further active malignancies, (iv) recorded patient characteristics as outlined in the section “Patient Demographics/-Characteristics, Follow-up and Clinical Data”.

### 2.2 PET/CT imaging

All [^18^F]FDG-PET/CT data were acquired on a Biograph mCT 128 (Siemens Healthineers, Erlangen, Germany). The average delay was 62 ± 5.17 min after injection of a bodyweight-adapted dosage of [^18^F]FDG (3 MBq/kg bodyweight). To ensure blood glucose levels below 150 mg/dL, blood samples were obtained, and patients needed to fast six hours prior to injection of [^18^F]-FDG. The mean activity applied to patients at t_0_ [^18^F]FDG-PET/CT was 230 ± 38 MBq, at t_1_ [^18^F]FDG-PET/CT was 241 ± 44 MBq, and at t_2_ [^18^F]FDG-PET/CT was 237 ± 46 MBq. PET/CT was performed with a total-body scan (14 / 48; 29 %) or a whole-body scan (34 / 48; 71 %). Weight-adapted iodinated contrast medium (Accupaque 300, GE Healthcare) was used in 21 / 48 (44 %) [^18^F]FDG-PET/CT scans in those patients without prior diagnostic (contrast-enhanced) whole-body CT. CT acquisition started 70 seconds after intravenous injection of the contrast agent. Automated tube current modulation was activated in all scans (presets 120 kV, 190 reference mAs, collimation 128 × 0.6 mm, pitch 0.8, slice thickness 1.5 mm). An additional diagnostic low-dose lung tissue scan in deep inspiration was added to all [^18^F]FDG-PET/CT to improve pulmonary imaging [24]. PET data were acquired for 3 min in each bed position (matrix size 200 × 200, axial field of view 21.8 cm and a Gaussian filter of 2.0 mm). Attenuation correction was performed and iterative reconstruction using ordered subsets expectation maximization was used with the following presets: 4 iterations and 8 subsets.

### 2.3 Image analysis

A board-certified radiologist experienced in nuclear medicine diagnostics and a board-certified nuclear medicine physician did further data evaluation of the acquired [^18^F]FDG-PET/CT datasets using a dedicated PACS-Workstation (IDS7; Sectra). The five-point (Deauville) scale for interpretation of [^18^F]FDG PET and the revised staging and response criteria of the LC were critically reviewed by both readers according to the available [^18^F]FDG-PET/CT findings to determine complete response (CR), partial response (PR), stable disease (SD), or PD. Potential disagreements were solved in consensus by reviewing all available clinical and imaging data.

Readers evaluated all data sets for lesions (lymphonodal / extranodal) suspicious of lymphoma. For lesion characterization on [^18^F]PET, visually increased focal FDG-uptake in comparison to background and mediastinum and higher than liver activity were considered indicative for involvement with active lymphoma in concordance with the five-point scale of the DS [25].

**Fig. 1:**
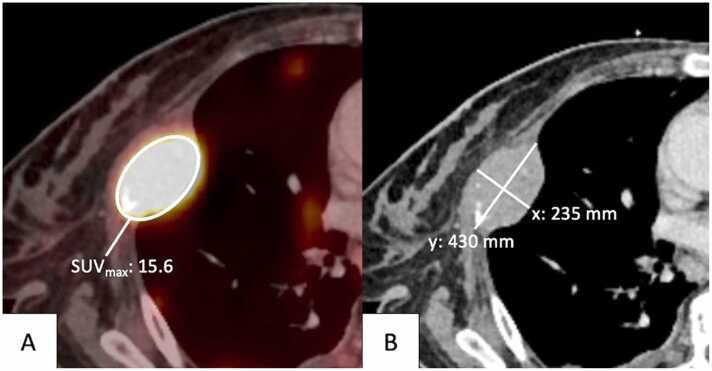
[^18^F]FDG-PET/CT examination of a fifty-one-year-old patient with DLBCL and pleural lymphoma manifestation and infiltration of adjacent rib. Example of image analysis with (**A**) measurement the SUV_max_ in the tumor area with the highest metabolic activity using a volume of interest (VOI), and (**B**) measurement of maximal (y) and minimal (x) diameters in mm.

The following morphologic and metabolic characteristics were collected on [^18^F]FDG-PET/CT for each patient to determine DS and stage according to LC: (i) maximum and minimum diameter of reference lesions in mm, (ii) tumor volume / SPD (sum of the product of diameters) in mm^2^ (iii), and SUV_max_ of the lesion with the highest metabolic activity using an area-adapted volume of interest (VOI). An example of data acquisition is visualized in Fig. 1. Focusing on the lymphoma manifestation with the highest metabolic activity, in analogy to previous studies measurement of the SUV_mean_ was omitted [26], [27]. For evaluation of DS, SUV_max_ of liver parenchyma and SUV_max_ of the mediastinal blood pool were measured for each patient in pre-defined regions.

### 2.4 Patient demographics/-characteristics, follow-up and clinical data

Patient demographics, clinical data (age, sex), Ann-Arbor stage [AAS], start- / endpoint of therapy, survival, and / or progression data were obtained from each patient. Survival / progression data were collected during clinical follow-up at t_1_ and at t_2_. Five-point DS was determined in each [^18^F]FDG-PET/CT. Response to therapy was defined in accordance to the revised staging and response criteria of the LC at t_1_ and t_2_ [25].

### 2.5 Statistical analysis

SPSS Statistics 26 (IBM Inc., Armonk, NY, USA) was used for statistical analysis. Descriptive analysis was performed, and data are presented as mean ± SD. For non-normally distributed continuous variables the median was reported including the interquartile range (IQR, 1st quarter - 3rd quarter). Correlations between [^18^F]FDG-PET/CT imaging parameters towards progression-free survival (PFS) and overall survival (OS) were examined by the Kaplan-Meier method. For PFS and OS the median period in days was specified, if the median OS was reached. The median was determined as a threshold value for SUV_max_ and SPD. Statistical significance was calculated using the log-rank test. Furthermore, cox regression and a hazard ratio (HR) were used to calculate the extent to which different [^18^F]FDG-PET/CT imaging markers or clinical parameters had a significant impact on PFS and OS. *P* values < 0.05 were set as statistically significant. For the hazard ratio, the corresponding 95 % confidence intervals (CI) were calculated.

## 3 Results

### 3.1 Patient characteristics and PET / CT metabolic and morphologic parameters

All 18 patients suffered from relapsed / refractory DLBCL. The median follow-up time was ten months (IQR 6–16) from CAR-T cell infusion. Twelve of 18 (67 %) patients had progressive disease and 6 / 18 (33 %) patients died within the study period. Six-month survival rate was 78 %. The objective response rate (ORR = CR + PR) was 45 % at t_1_ and t_2_. A detailed overview of patient demographics/-characteristics and CAR-T cell therapy drugs is given in Table 1**.**

**Table 1:** Detailed overview of patient demographics/-characteristics and CAR-T cell therapy drugs.

PATIENT DEMOGRAPHICS/-CHARACTERISTICS/CAR-T CELL THERAPY DRUGS	VALUE (PERCENTAGE)
**Number of patients**	n = 18
**Age (years)**	
Mean ± SD	60 ± 12
**Gender**	
Female	n = 8 / 18 (44 %)
Male	n = 10 / 18 (56 %)
**Initial Ann-Arbor stage (AAS)**	
I	n = 3 / 18 (17 %)
II	n = 1 / 18 (6 %)
III	n = 5 / 18 (28 %)
IV	n = 10 / 18 (56 %)
**CAR-T cell therapy**	
Axicabtagene ciloleucel (Yescarta®)	n = 11 /18 (61 %)
Tisagenlecleucel (Kymriah®)	n = 7 / 18 (39 %)
**Objective response rate (ORR)**	
t_1_	n = 8 (45 %)
t_2_	n = 8 (45 %)
**Progress within the study period**	
Yes	n = 12 / 18 (67 %)
No	n = 6 / 18 (33 %)
**Death within the study period**	
Yes	n = 6 / 18 (33 %)
No	n = 12 / 8 (66 %)

The median SPD at t_0_ was 2320 mm^2^ (IQR 500–4200), at t_1_ it was 980 mm^2^ (IQR 240–2220) and at t_2_ it was 1350 mm^2^ (IQR 560–3800). Furthermore, the median SUV_max_ at t_0_ was 9.0 (IQR 5.4–16.0; range: 1.8–37.0), at t_1_ it was 5.0 (IQR 2.0–9.0; range: 1.4–34.0) and at t_2_ it was 2.8 (IQR 2.0–6.5; range: 1.1–40.0).

### 3.2 Prediction of survival

**Fig. 2:**
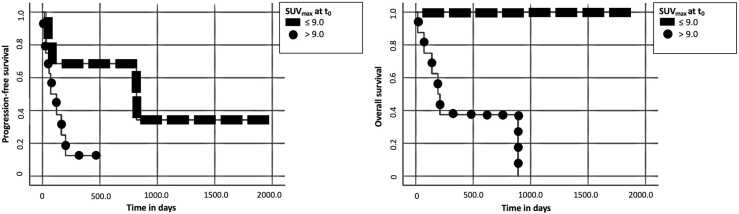
Kaplan-Meier curves of SUV_max_ at t_0_. Progression-free survival (left) and overall survival (right) proportion against time in days are plotted in each diagram.

PFS and OS differed significantly when applying an SUV_max_ threshold of 9.0 at t_0_ (PFS: *p* = 0.04; OS: *p* < 0.01). The median PFS of patients with an SUV_max_ > 9.0 was 71 days compared to 818 days for patients with an SUV_max_ ≤ 9.0 at t_0_. Median OS was 193 days for patients with an SUV_max_ > 9.0 at t_0_, for patients with SUV_max_ ≤ 9.0 the median OS was not reached. Kaplan-Meier curves for PFS and OS of SUV_max_ at t_0_ are visualized in Fig. 2.

SUV_max_ > 9.0 at t_0_ and SUV_max_ > 5.0 at t_1_ were associated with an increased risk of PD, but there was no statistically significant difference when comparing the threshold-associated groups concerning death. A DS > 3 at t_1_ was associated with an increased risk of a PD without significant elevated risk of death (see Table 2).

**Table 2:** Overview of univariate analysis of pre- / post-CAR-T [^18^F]FDG-PET/CT variables for progression-free survival (PFS) and overall survival (OS).

	PFS *P* HR (95 % CI)	OS *P* HR (95 % CI)
**PET/CT data at t**_**0**_		
SUV_max_	0.17 1.0 (0.9–1.1)	0.14 1.0 (0.9–1.1)
SUV_max_ > 9.0	0.03* 7.0 (1.3–40.5)	0.18 12.3 (0.1–14.8)
SPD	0.19 1.0 (0.9–1.0)	0.06 1.0 (1.0–1.0)
SPD > 2320 mm^2^	0.28 1.9 (0.6–6.1)	0.21 4.1 (0.5–37.0)
**PET/CT data at t**_**1**_		
SUV_max_	0.15 1.1 (1.0–1.1)	0.55 1.0 (0.9–1.1)
SUV_max_ > 5.0	< 0.01* 7.8 (1.9–32)	0.35 2.4 (0.4–14.3)
SPD	0.05 1.0 (1.0–1.0)	0.13 1.0 (1.0–1.0)
SPD > 980 mm^2^	0.93 1.1 (0.3–3.5)	0.54 0.6 (0.1–3.9)
DS > 3	0.04* 8.2 (1.1 – 61.3)	0.24 2.8 (0.5–15.8)
Lugano PD	0.02* 5.0 (1.2–20.5)	< 0.05* 5.0 (1.1–26.0)
**PET/CT data at t**_**2**_		
SUV_max_	0.15 1.0 (1.0–1.0)	0.98 1.0 (0.9–1.0)
SUV_max_ > 2.8	0.65 1.3 (0.4–4.7)	0.56 1.7 (0.3–10.6)
SPD	0.09 1.0 (1.0–1.0)	0.03* 1.0 (1.0–1.0)
SPD > 1350 mm^2^	0.33 2.0 0.5–8.0)	0.38 66.3 (0.1–72 000)
DS > 3	0.30 1.0 (0.5–8.0)	0.29 51.4 (0.0–79 500)
Lugano PD	0.01* 8.0 (1.6–40.1)	0.10 6.0 (0.8–54.3)

**Notes:** SUV_max_ and SPD were also analyzed as dichotomous variables, applying medians as cut-offs. *P*-values and hazard ratios (HR) with 95 % confidence interval (CI) are presented. * indicates statistical significance. SPD: sum of the product of diameters. DS: Deauville score. Lugano PD: progressive disease according to Lugano classification.

**Fig. 3:**
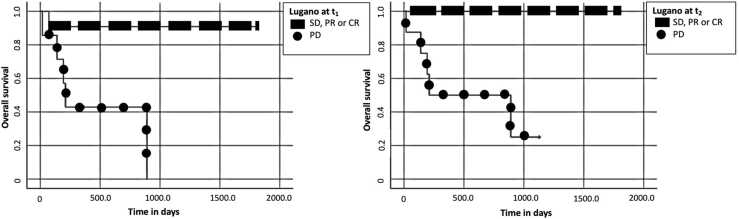
Kaplan-Meier curves of Lugano classification at t_1_ (left) und t_2_ (right): Complete response (CR), partial response (PR), stable disease (SD) and progressive disease (PD). Overall survival proportion against time in days are plotted in each diagram.

A PD according to LC at t_1_ and t_2_ was associated with a significantly shorter OS (t_1_: *p* = 0.02, median OS: 211 days versus not reached; t_2_: *p* < 0.01, median OS: 891 days versus not reached) towards patients with CR, PR or SD, using the Kaplan-Meier method. Patients with PD according to LC at t_1_ had an increased risk of death (see Table 2). Kaplan-Meier curves for OS of LC are visualized in Fig. 3.

## 4 Discussion

There is only limited data available on the role of [^18^F]FDG-PET/CT in CAR-T cell therapy. To deepen the understanding, this study aimed to investigate the value of [^18^F]FDG-PET/CT in relapsed / refractory DLBCL-patients undergoing CAR-T cell therapy, especially examining its prognostic potential. This study suggests a role of [^18^F]FDG-PET/CT both, in defining the patients’ prognoses prior to therapy and in differentiating therapy response from therapy failure.

According to the presented data, especially the pre-therapeutically evaluated SUV_max_ may play a pivotal role when predicting response to CAR-T cell therapy. Our data indicate that refractory / relapsed DLBCL-patients have a higher risk for a poor course of disease (PFS, OS) if pre-therapeutic SUV_max_ exceeds 9.0. Thus, easily accessible SUV evaluation during clinical routine could have a decisive impact on therapeutic decision making for DLBCL-patients, which can be supported by already existing studies [22], [28], [29]. Cohen et al. [22] demonstrated that pre-therapeutic SUV_max_ can guide patient’s selection for CAR-T cell therapy and A. Al Zaki et al. [28] indicated increased risk of PD one month after CAR-T cell infusion if pre-therapeutic SUV_max_ exceeds 10.0 [22], [28]. The data evaluation of Lacoboni et al. in 2021 revealed no significant correlation of SUV_max_ values towards PFS [17]. However, the calculated SUV_max_ threshold of 20.0 by far exceeds the presented threshold as well as the threshold of Al Zaki et al. [28] which aggravated the comparability of study results. In this context, it is important to acknowledge that both, our study and already existing research often feature relatively brief follow-up periods and small patient populations due to the novelty of CAR-T cell therapy.

Furthermore, our data have demonstrated patients with a pre-therapeutic SUV_max_ > 9.0 to have an increased risk of PD but not an elevated risk of mortality. This discrepancy could be attributed to the small patient cohort, the low number of deaths, or be influenced by the magnitude of patient's tumor burden. Nonetheless, it indicates a worse course of disease as already explained.

SUV_max_ values are mandatory in the determination of DS, a clinically established reporting scale for assessing therapeutic response in lymphoma patients undergoing therapy [13], [30]. Consistent with findings by Cohen et al. [22], a DS > 3 at t_1_ was associated with early therapy failure demonstrating a significantly higher risk of PD compared to patients with a DS ≤ 3. Cohen et al. [22] identified a DS > 3 as the strongest prognostic predictor for OS and A. Guidetti et al. [31] explained according to their results that patients with a DS > 3 had a worse one-year survival than patients with a DS of 1–3 [22], [31]. Our study did not find a significantly higher risk of death in patients with higher DS. Comparable outcomes have been observed by Kuhnl et al. (2022). In their study, the difference in PFS across DS stages was markedly significant, while the impact on OS was only modest [30]. In addition to DS, LC serves as another clinically established tool for evaluating treatment response in DLBCL-patients. Early detection of treatment failure is necessary for prompt treatment adjustments of patients undergoing CAR-T cell therapy [25]. Patients exhibiting PD according to LC one month after CAR-T cell infusion had a significant shorter OS compared to patients with a CR, PR or SD. Although our analysis covers short time intervals, it suggests that PD about one month after CAR-T cell infusion may be a relevant clinical parameter linked to diminished OS. This aligns with findings from Georgi et al. (2023), who reported significantly better outcomes for patients with CR [12]. In clinical routine an integration of both, DS and LC in the post-CAR-T [^18^F]FDG-PET/CT diagnostic report appears to be helpful for therapeutic decision making. Particularly, the [^18^F]FDG-PET/CT scan one month after CAR-T cell infusion could be suitable in distinguishing between responders and non-responders. This capability facilitates early adjustments to treatment protocols, contributing to a more refined and patient-centered therapeutic approach [11].

There are certain limitations to our study. The retrospective study design is the first limitation, as selection bias and further confounding factors cannot be excluded. The second and third limitation stems from the small patient cohort and the short follow-up time of ten months, reflecting the current clinical reality of available data. However, precisely due to the limited number of patients undergoing CAR-T cell therapy and the sporadic availability of studies on this topic, it is highly important to analyze early data for patient-centered therapeutic improvement. Additionally, it must be noted that at our department, only one pre-CAR-T [^18^F]FDG-PET/CT was conducted, unlike at other locations where two are typically performed, pre-leukapheresis and pre-lymphodepletion. To further enhance the understanding of CAR-T cell therapy at both, imaging and molecular levels, further homogeneous, multicenter studies are essential to refine patient treatment approaches.

## 5 Conclusion

SUV_max_ of pre-CAR-T [^18^F]FDG-PET/CT could be a suitable predictor for risk stratification of relapsed / refractory DLBCL-patients. Additionally, DS and LC may serve as response tools for identifying early therapy failure in patients with relapsed / refractory DLBCL undergoing CAR-T cell therapy.

## CRediT authorship contribution statement

**Peters Helena Anne:** Writing – original draft, Visualization, Validation, Investigation, Formal analysis, Data curation. **Guido Kobbe:** Writing – review & editing, Supervision, Resources. **Aleksandar Radujkovic:** Writing – review & editing, Data curation. **Eduards Mamlins:** Writing – review & editing, Data curation. **Kai Jannusch:** Writing – review & editing, Visualization, Supervision, Methodology, Investigation, Conceptualization. **Gerald Antoch:** Writing – review & editing, Supervision, Resources. **Johannes Fischer:** Writing – review & editing, Data curation. **Sascha Dietrich:** Writing – review & editing, Supervision, Resources. **Nora Liebers:** Writing – review & editing, Data curation. **Frederik L. Giesel:** Writing – review & editing, Supervision, Resources. **Vivien Lorena Ivan:** Writing – review & editing, Data curation. **Christina Antke:** Writing – review & editing, Data curation. **Matthias Boschheidgen:** Writing – review & editing, Data curation. **Paul Jäger:** Writing – review & editing, Data curation. **Daniel Weiss:** Writing – review & editing, Data curation. **Kathrin Nachtkamp:** Writing – review & editing, Data curation. **Emil Novruzov:** Writing – original draft, Visualization, Validation, Investigation, Conceptualization. **Peter Minko:** Writing – review & editing, Data curation. **Ben-Niklas Bärmann:** Writing – original draft, Visualization, Validation, Investigation, Formal analysis, Data curation. **Julian Kirchner:** Writing – review & editing, Data curation.

## Declaration of Generative AI and AI-assisted technologies in the writing process

During the preparation of this work the authors used Deepl and ChatGPT for writing and content editing. After using this tool/service, the authors reviewed and edited the content as needed and take full responsibility for the content of the publication.

## Informed consent

Written informed consent was waived by the Institutional Review Board.

## Ethical statement

All procedures performed in studies involving human participants were in accordance with the ethical standards of the institutional research committee (Heinrich-Heine university, university hospital Düsseldorf) and with the 1964 Helsinki declaration and its later amendments or comparable ethical standards (date of acceptance 29.11.2023; study number: 2023–2618).

## Ethical approval

Institutional Review Board approval was obtained (Heinrich-Heine university Duesseldorf; study number 2023–2618; date of approval: 29/11/2023).

## Funding

This research did not receive any specific grant from funding agencies in the public, commercial, or not-for-profit sectors.

## Declaration of Competing Interest

The authors declare that they have no known competing financial interests or personal relationships that could have appeared to influence the work reported in this paper.

## Data Availability

Data cannot be shared publicly because of data protection regulations in Germany and the requirements of the ethics committee. Data are available from the institutional research committee Duesseldorf Heinrich-Heine university (contact via study number 2023–2618) for researchers who meet the criteria for access to confidential data.
